# Gastric coconut bezoar following Roux-en-Y gastric bypass

**DOI:** 10.1093/jscr/rjag357

**Published:** 2026-05-11

**Authors:** Silvana Alexandra Valencia Valverde, Fernando Xavier Moyón Constante, Sara Paola Loaiza Romero

**Affiliations:** Department of Bariatric Surgery, General Hospital San Francisco, Quito 170120, Ecuador; Department of Bariatric Surgery, General Hospital San Francisco, Quito 170120, Ecuador; Department of Anesthesiology, Falconí Clinical Center, Quito 170120, Ecuador

**Keywords:** Roux-en-Y gastric bypass, coconut bezoar, gastrointestinal obstruction, bariatric endoscopy, phytobezoar

## Abstract

Coconut bezoars are rare causes of gastrointestinal obstruction, particularly in patients who have undergone Roux-en-Y gastric bypass (RYGB). Altered gastric motility and anatomical changes after bariatric surgery predispose patients to the accumulation of indigestible fibers. We report the case of a 45-year-old female who presented one year after RYGB with nausea, vomiting, and abdominal pain. Upper endoscopy revealed a coconut bezoar in the gastric pouch, which was successfully treated with endoscopic mechanical fragmentation and extraction. The patient recovered fully without surgical intervention. This case highlights the importance of considering bezoars in post-bariatric patients presenting with obstructive gastrointestinal symptoms and demonstrates the effectiveness of endoscopic management.

## Introduction

A bezoar is a rare but recognized cause of gastrointestinal obstruction, particularly in patients with previous gastric surgery. Phytobezoars, composed of indigestible plant fibers, are the most common type and may develop in individuals with altered gastrointestinal anatomy or motility. Coconut bezoars, formed from undigested coconut fibers, are particularly uncommon and may present a diagnostic and therapeutic challenge. The anatomical and physiological alterations following Roux-en-Y gastric bypass (RYGB) can predispose patients to bezoar formation due to changes in gastric motility, decreased gastric acid secretion, and altered digestion [1, 2].

## Case report

**Figure 1:**
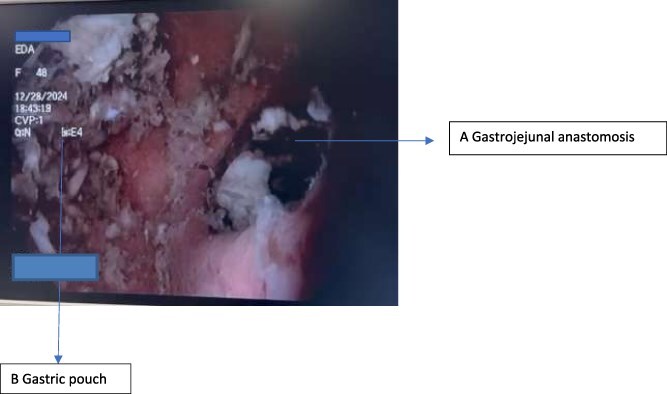
Endoscopic view of the coconut bezoar located within the gastric pouch.

A 45-year-old female patient with a history of RYGB performed one year prior presented with nausea, vomiting for 48 hours, abdominal pain, and a sensation of fullness. Given her surgical history, upper gastrointestinal obstruction was suspected. Endoscopic evaluation confirmed the presence of a coconut bezoar in the gastric pouch (Fig. 1). Endoscopic treatment was performed using mechanical fragmentation with a basket and forceps to facilitate extraction. The procedure was successful, and the patient’s symptoms resolved without the need for surgical intervention. The patient was discharged with dietary counseling and was advised to avoid high-fiber, poorly digestible foods to prevent recurrence.

## Discussion

Bezoars are uncommon but well-documented complications following gastric surgery, including bariatric procedures. Previous gastric surgery is considered one of the main risk factors for bezoar formation due to alterations in gastric anatomy, motility, and acid secretion [1, 3].

Globally, bezoars are considered rare, with an estimated incidence of ~0.3% in the general population. In cases of gastrointestinal obstruction, bezoars account for ~0.4%–4% of cases [4]. The risk is increased in patients with previous gastric surgery, with some studies reporting that 20%–93% of bezoar cases occur in patients with a history of gastric operations [5].

Patients who undergo RYGB may be particularly predisposed to bezoar formation due to reduced gastric capacity, delayed gastric emptying, and altered dietary habits. Additionally, reduced gastric acidity and impaired mechanical digestion can facilitate the accumulation of indigestible plant fibers within the gastric pouch. Coconut fibers, due to their high cellulose content and resistance to enzymatic digestion, may contribute to bezoar formation in susceptible individuals.

Diagnosis is most effectively established through upper gastrointestinal endoscopy, which allows both direct visualization and therapeutic intervention. Endoscopic removal is generally considered the first-line treatment, as it offers a minimally invasive approach with high success rates [6]. However, in cases of large, impacted, or complicated bezoars, surgical intervention may be required.

This case emphasizes the importance of maintaining a high index of suspicion for bezoars in post-bariatric surgery patients presenting with obstructive gastrointestinal symptoms. Early diagnosis and endoscopic treatment can prevent complications and avoid the need for surgical management. Furthermore, nutritional education and dietary modification remain essential preventive strategies to reduce the risk of recurrence in this patient population.
